# Linking Global HIV/AIDS Treatments with National Programs for the Control and Elimination of the Neglected Tropical Diseases

**DOI:** 10.1371/journal.pntd.0001022

**Published:** 2011-07-26

**Authors:** Julie Noblick, Richard Skolnik, Peter J. Hotez

**Affiliations:** 1 School of Public Health and Health Sciences, The George Washington University, Washington, D.C., United States of America; 2 Department of Global Health, School of Public Health and Health Sciences, The George Washington University, Washington, D.C., United States of America; 3 Sabin Vaccine Institute – Texas Children's Hospital – Baylor College of Medicine – Center for Vaccine Development, Houston, Texas, United States of America; 4 Departments of Pediatrics and Molecular Virology & Microbiology, and National School of Tropical Medicine, Baylor College of Medicine, Houston, Texas, United States of America

*Emerging evidence provides a scientific rationale for combining treatment programs for neglected tropical diseases (NTDs) with programs for the treatment of HIV/AIDS. Engaging the major stakeholders to establish operational links between HIV/AIDS and NTD control and elimination activities, especially in sub-Saharan Africa, could increase the efficiency and cost-effectiveness of both HIV/AIDS and NTD efforts.*

## NTDs in Sub-Saharan Africa

Sub-Saharan Africa is home to more than 90% of the world's cases of onchocerciasis and schistosomiasis, about one-half the world's lymphatic filariasis and trachoma, and one-third of all global soil-transmitted helminth infections [1]. These infections exhibit extensive geographic overlap and polyparasitism in Africa is extremely common [1]–[3]. The neglected tropical diseases (NTDs) produce a devastating level of chronic disability in sub-Saharan Africa, with some estimates suggesting that the NTD disease burden exceeds tuberculosis and is one-half that of malaria [1]. Most of the African population living in poverty is infected with one or more NTDs [1]. In children, the NTDs are responsible for anemia and other forms of malnutrition, intestinal obstruction, and impaired physical and cognitive development [2]–[4]. In adults, NTDs cause anemia, especially in pregnancy, damage to internal organs, and physical disfigurement [2]–[4]. The NTDs are among the most common infections affecting the health and well-being of girls and women [5]. Through their impact on child development, pregnancy outcome, and worker productivity, the NTDs also thwart economic development [6].

Because of the availability of donated or generically available inexpensive drugs, it is possible through the mass drug administration (MDA) of “rapid impact” packages containing albendazole/mebendazole, ivermectin, praziquantel, and azithromycin to reduce the prevalence, and in some cases either control or eliminate these NTDs, for as little as US$0.50 per person annually [6]. MDA for the soil-transmitted helminth infections and schistosomiasis, Africa's highest prevalence NTDs, has been priced at US$0.32 per person annually [7]. As a result of these low-cost and highly cost-efficient opportunities for what is sometimes referred to as *preventive chemotherapy*, the United States Agency for International Development (USAID) is supporting national programs of control or elimination of the most common NTDs in multiple African countries [8], with additional country-wide programs expected to be announced in the coming year. Additional national programs for scale-up of NTD control are being supported by the British Department for International Development (DFID) [9], while the Global Network for Neglected Tropical Diseases is mobilizing private resources for Rwanda and Burundi, as well as other countries [3], [6].

## HIV/AIDS and NTD Co-Infections

HIV/AIDS and NTD co-infections are widespread in sub-Saharan Africa, and there is evidence that the high prevalence helminth infections, i.e., the three major soil-transmitted helminth infections (ascariasis, hookworm, trichuriasis), and schistosomiasis either promote susceptibility to the HIV virus or worsen the clinical course and progression of AIDS, while visceral leishmaniasis (VL) has emerged as an important opportunistic infection of HIV/AIDS.

### Visceral Leishmaniasis and HIV

The relationship between HIV/AIDS and NTDs has been noted since the early 1990s. The World Health Organization has recognized that VL can present as an aggressive opportunistic infection in people living with HIV/AIDS (PLWHA). If left untreated, VL alone will cause mortality rates as high as 100% within two years [10]. In co-infected individuals, VL and HIV mutually influence one another's disease progression, causing unrestrained pathogen reproduction and profound immunosuppression, and higher VL treatment failures and relapses [10]. Because VL and HIV co-infections occur predominantly in remote rural areas of East Africa, there is little available data on the prevalence of this problem [11]. However, in northwest Ethiopia, up to 30% of all VL patients are believed to be co-infected with HIV [11]. Similar opportunistic associations between other NTDs, such as Chagas disease, may occur in patients with HIV/AIDS [12].

### Helminth Infections and HIV

The geographic overlap between the high prevalence helminth infections and HIV/AIDS in sub-Saharan Africa is extensive. More than 100 million people in sub-Saharan Africa are infected with ascariasis, hookworm, trichuriasis, or some combination of these soil-transmitted helminth infections [1]; additional estimates suggest that more than 400 million people suffer from schistosomiasis [13]. The geographic overlap is particularly striking between urogenital schistosomiasis (*Schistosoma haematobium* infection) and HIV/AIDS in the large southern and East African countries of Kenya, Mozambique, South Africa, Tanzania, Zambia, and Zimbabwe, and to some extent, Cameroon in West Africa [7], [14].

**Figure 1:**
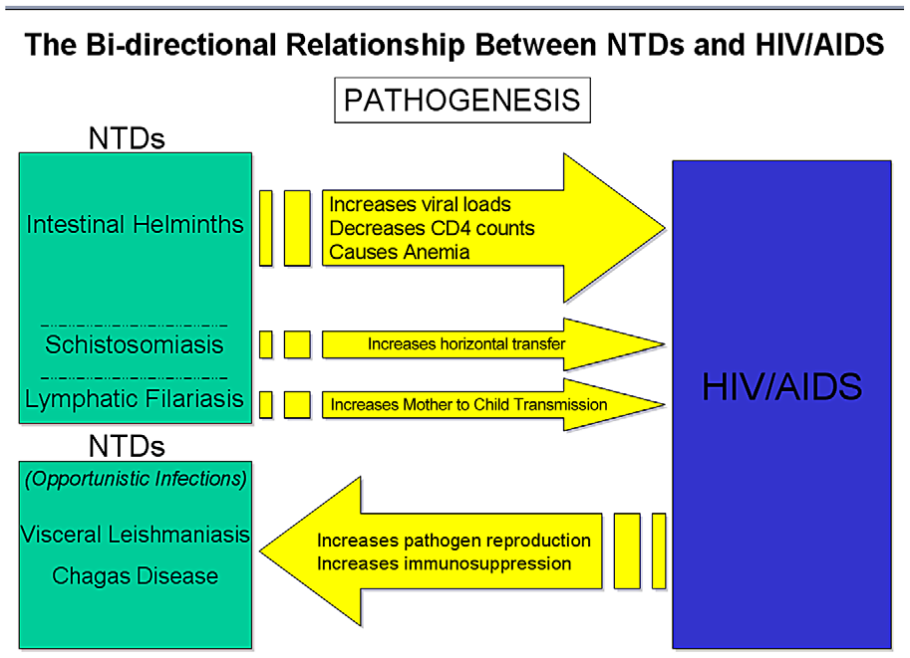
Schematic diagram highlighting the bi-directionality of the pathogenic links between NTDs and HIV/AIDS.

These high prevalence helminth infections have an adverse, albeit largely hidden, impact on the AIDS epidemic in Africa [2], [14]–[21]. While there are conflicting data on this front [22]–[24], a systematic review of randomized clinical trials demonstrates the beneficial effects of deworming in terms of reduced HIV viral loads and/or elevations in CD4 counts [25]. Still further evidence suggests that maternal helminth infections promote maternal-to-child transmission of HIV/AIDS, possibly as a result of increased maternal HIV viral loads [26]. These effects were summarized recently [14]. As a plausible mechanism, one unifying hypothesis suggests that helminth infections are immunomodulatory, possibly diminishing host innate immunity to HIV to promote viral replication and T cell diminution [2], [15]–[21]. Equally compelling are data from Zimbabwe that female genital schistosomiasis (FGS), occurring in up to 75% of women with *S. haematobium* infection, increases the risk of humans acquiring HIV infection 3-fold [27]. Studies are underway elsewhere in southern and East Africa to confirm these results. There is also evidence that acute schistosomiasis from *Schistosoma mansoni* infection increases the risk of new HIV acquisition in rhesus macaques [28]. These studies, together with the observation that most of the poorest people who live in sub-Saharan Africa below the World Bank poverty level are infected with schistosomes or some other helminths [1], [13], suggest that helminth infections may represent a potent force fueling Africa's AIDS epidemic [29]. Therefore, early intervention to prevent FGS through MDA with praziquantel for young girls may represent a highly cost-effective HIV/AIDS prevention strategy [4], [5], [7] (Figure 1).

## Linking Existing HIV Initiatives with NTD Control

The high prevalence of helminth infections and other NTDs in sub-Saharan Africa and their medical significance in terms of affecting Africa's AIDS epidemic demand a public health response from the established global HIV/AIDS community, in parallel with efforts to scale up NTD control. At the recent United Nations 2010 Millennium Development Goals Summit, US President Barack Obama announced a new approach to the programs that make up the United States government's Global Health Initiative. At the core of this new directive is a call for the integration of programs and investments across health priorities [30].

We support the US President's call for integration and would favor strategic planning to examine how the US President's Emergency Plan for AIDS Relief (PEPFAR) and the Global Fund to Fight AIDS, Tuberculosis and Malaria (GFATM), the two largest global programs committed to delivering anti-retroviral drugs and other AIDS prevention measures, could advance efforts to coordinate and link the HIV/AIDS programs they support with NTD control and elimination programs [14]. Additional links with programs for malaria and tuberculosis control have also been proposed [2], [14].

As noted above, national programs of NTD control and elimination are underway in more than a dozen sub-Saharan Africa countries, with additional programs expected in the coming years. These NTD preventive chemotherapy programs deliver partial or complete rapid impact packages, together with environmental control measures or simple surgeries, under the auspices of public–private partnerships working with national health ministries [3], [4], [6]. NTD control and elimination is conducted through a variety of mechanisms, including community-based drug distribution and school-based drug distribution by teachers or through child health days [3], [4], [6].

**Figure 2:**
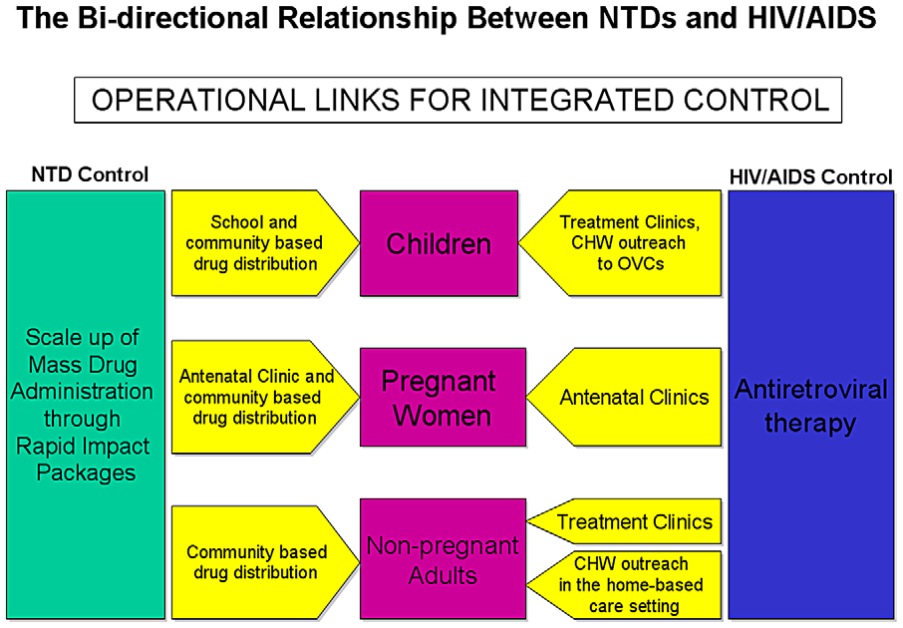
Schematic diagram representing the operational links for integrated control of NTDs and HIVS/AIDS.

Currently, both PEPFAR- and GFATM-supported programs provide treatment for deadly tuberculosis co-infections in PLWHA [31], as well as co-trimoxazole treatment to prevent opportunistic infections. They also provide nutritional support for PLWHA. We propose that PEPFAR- and GFATM-supported programs could also work with USAID's Neglected Tropical Disease Program and similar initiatives supported by DFID and the Global Network for NTDs to mitigate the impact of NTD-related morbidity on their target populations [14]. Such NTD control programs could continue to operate autonomously, but coordinate the scale-up of MDA interventions in areas found to bear high prevalence rates of NTDs and HIV/AIDS. In addition to scaling up MDA efforts, PEPFAR- and GFATM-assisted programs could engage in targeted interventions in response to evidence that NTD control could both decrease susceptibility to HIV infection and improve morbidity levels in seropositive individuals. Here, we use some of the programs of PEPFAR as examples to illustrate how NTDs might fit into an existing framework for the care and treatment of HIV/AIDS (Figure 2).

## Treatment of NTD Co-Infections

In terms of providing treatment, there are several areas within PEPFAR's programming that could link with NTD control efforts. Just as PEPFAR programs provide treatment options for tuberculosis co-infections and chemoprophylaxis to prevent other opportunistic infections, NTD co-infections for PLWHA that receive treatment through PEPFAR clinics could also be addressed. These facilities could be stocked with appropriate NTD medications based on regional NTD prevalence. This approach would allow PLWHA to be treated at the point of service, thus minimizing the impact of NTDs on viral replication. In addition, because many individuals who live in NTD-endemic areas are unaware of their HIV status, NTD control and elimination campaigns could be expanded to provide HIV testing and care. In this way there could be mutual and bi-directional benefit.

PEPFAR antenatal clinics could actively engage in NTD control as well. Up to one-third of pregnant women in sub-Saharan Africa are infected with hookworms [32], while large numbers of pregnant women are likely infected with schistosomiasis [33], and suffer from severe anemia as a result [1], [32], [33]. All pregnant mothers visiting PEPFAR antenatal clinics in their second or third trimesters could receive single doses of albendazole and praziquantel to prevent morbidity caused by soil-transmitted helminth or schistosome infections, respectively. Given that schistosome infection in young girls may cause FGS in childhood prior to sexual debut [7], [27], thus increasing susceptibility to HIV infection, PEPFAR could cooperate with existing NTD control programs to expand its targeted prevention efforts for women beyond just antenatal clinics, especially for the targeted treatment of FGS.

### Care

PEPFAR's care initiatives have two entry points for NTD control, both of which would decrease morbidity in PLWHA by building upon existing programming. PEPFAR community health care workers (CHWs) visit the homes of orphans and vulnerable children (OVC) infected and affected by HIV/AIDS to monitor growth and development. These health care workers can be trained to administer the appropriate NTD chemoprophylaxis based on prevalence mapping. Many OVCs, who are often missed by school-based deworming programs, would benefit. Similar to the OVC program, PEPFAR sends CHWs to the homes of PLWHA that are unable to travel to local clinics. PEPFAR could integrate NTD control into both the OVC and home-based care programs to respond to the risk of chronic immune activation in co-infected individuals.

### Research

Operational research to evaluate the successes and failures for HIV/AIDS and NTD control are critical, as is research and development for new and improved control tools for the NTDs [34].

## Integration in Action

Integrating NTD control into a PEPFAR or GFATM framework would require careful coordination and collaboration with the USAID's NTD Program, as well as with parallel efforts supported by DFID and the Global Network for NTDs, and most importantly the health ministries in the disease-endemic countries together with public–private partnerships committed to NTD control. Embracing the control of the NTDs, the most common infections of girls and women in developing countries [5], is also consistent with recent calls to expand PEPFAR's mandate to include maternal and child health initiatives [35]. In the meantime, there is a need for studies to confirm the cost-effectiveness of linking NTDs with HIV/AIDS. Funding will need to be arranged for the proposed scale-up of MDA by USAID's NTD Program and targeted interventions, as carried out by PEPFAR and GFATM. However, with the exception of praziquantel, most NTD drugs are fully funded through existing public–private partnerships with pharmaceutical companies, meaning that the overall costs will be relatively low, particularly as compared to the cost of intervening for malaria or tuberculosis [14].

Evidence speaking to the biological relationship between NTDs and HIV/AIDS means that effective management of HIV/AIDS may require the control of NTDs. The past successes in integrating directly observed therapy for tuberculosis [31], co-trimoxazole chemoprophylaxis, and nutritional support into HIV/AIDS programming indicates it is also possible to incorporate NTD preventive chemotherapy in HIV/AIDS treatment regimens. A full consideration of NTD control will require meaningful cooperation from the public health community. Bilateral organizations will need to nurture systems of collaboration within their existing operational frameworks. Multilateral and private funding organizations will need to promote the programmatic integration of control and actively seek out organizations prepared to implement new strategies. Like any public health initiative requiring a systems change on a global scale, change has been slow to arrive. However, as the consequences of decades of neglect accumulate, and the AIDS pandemic itself continues, it is more and more apparent that the public health community can no longer afford to disregard the importance of aggressively controlling NTDs as part of a strategy to address HIV infection.
